# Effects of rotation on temperature fluctuations in turbulent thermal convection on a hemisphere

**DOI:** 10.1038/s41598-018-34782-0

**Published:** 2018-11-08

**Authors:** T. Meuel, M. Coudert, P. Fischer, C. H. Bruneau, H. Kellay

**Affiliations:** 1 Université de Bordeaux, Laboratoire Ondes et Matière d’Aquitaine, UMR 5798 du CNRS, 351 cours de la Libération, 33405 Talence, France; 2 Université de Bordeaux, Institut de Mathématiques de Bordeaux, UMR 5251 du CNRS, INRIA Bordeaux - Sud Ouest Team MEMPHIS, 351 cours de la Libération, 33405 Talence, France; 3 Present Address: OIST, Okinawa, Japan

## Abstract

Rotation is present in many physical and geophysical systems and its role in determining flow properties and modifying turbulent fluctuations is of crucial importance. Here we focus on the role of rotation on temperature fluctuations in turbulent thermal convection. The system used consists of a rotating half soap bubble heated from below. This system has features, curvature and a quasi two dimensional character, which are reminiscent of atmospheric and planetary systems. Our experiments and numerical simulations show that rotation changes the nature of turbulent fluctuations and a new scaling regime is obtained for the temperature field. This change in the scaling behavior of temperature fluctuations, due to rotation, is put forth by studying the so called second moment of temperature differences across different scales. For high enough rotation rates, these temperature differences display a transition from Bolgiano Obukhov scaling to a new scaling regime. This scaling is at odds with expectations from theory, numerics, and experiments in three dimensions, suggesting that the effects of rotation on turbulent flows depend strongly on geometry and spatial dimension.

## Introduction

Turbulent thermal convection is important for numerous processes of meteorological, geophysical, and industrial interest. Several experimental and theoretical studies have focused on the properties of this turbulent state^1,2^ in the classical situation of a fluid enclosed in a container subject to a controlled temperature gradient^3^. As for hydrodynamic turbulence^4–6^, large fluctuations of the temperature and of the velocity of the fluid characterize turbulent thermal convection. These fluctuations can be described by using scaling laws which, according to^7,8^, are of the Kolmogorov type while, according to others^9^, are similar to those suggested for stably stratified convection^9–11^. Several experiments have measured these statistical properties but a number of issues regarding these scaling laws, and notably whether the statistics are Kolmogorov like or Bolgiano like, remain unresolved^9^. Recently, two dimensional (2D) turbulent thermal convection has been demonstrated in either vertical soap films^12–14^ or soap bubbles^15,16^. A detailed examination of the statistical properties of the velocity fluctuations, the density variations^12–15^, as well as the temperature fluctuations^16^ showed that they displayed scaling laws predicted by Bolgiano and Obukhov in the 1950’s for turbulence in stably stratified media^9–11,17–20^. The presence of such scaling laws as well as the ease of measuring the spatial variations of different quantities (velocity, temperature and density variations) make such two dimensional versions ideal models for the study of scaling and self similarity of turbulent flows governed by Bolgiano-Obukhov scalings.

**Figure 1:**
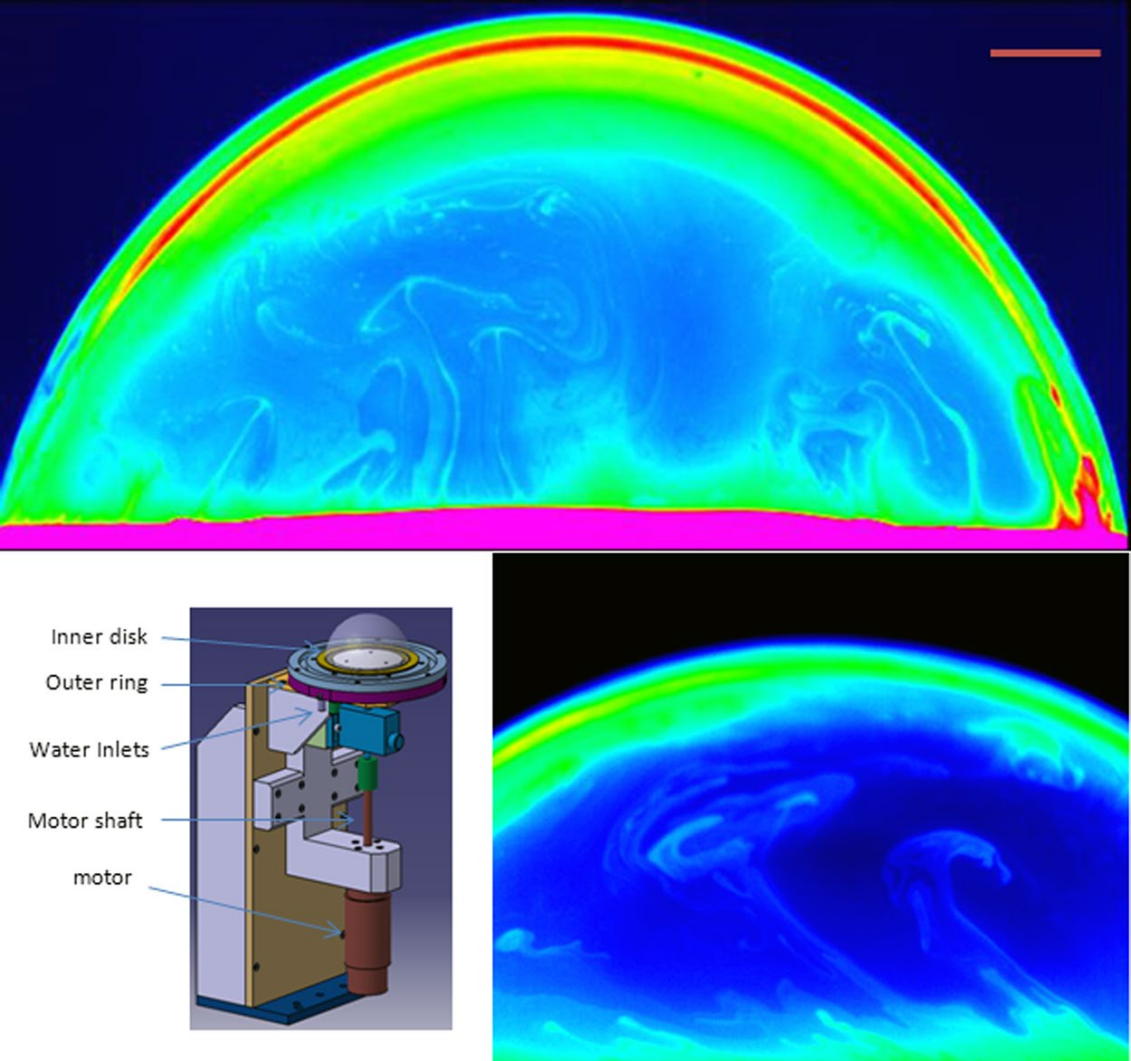
The schematic shows the experimental set up used. Infrared images of the bubble: top: Δ*T* = 16 °C and no rotation, the periphery of the image suffers from aberrations due to the curvature, bottom: Δ*T* = 16 °C for a rotation rate of 1.8 Hz, the scale bar is 1 cm in length and applies to both images.

Here, we investigate the role of rotation on the statistical properties of the temperature fluctuations, a scalar field, in a half soap bubble heated from below. Global rotation is inherent to a number of geophysical and planetary processes but how it affects scalar quantities such as the concentration of pollutants, the humidity or the temperature remains without experimental support. The examination of the role of rotation on the velocity fluctuations in three dimensional (3D) hydrodynamic turbulence has been shown to introduce novel features and changes in scaling behavior in experiments and simulations^21–24^. This change in scaling for the velocity field has direct consequences on the scaling of scalar fields in rotating turbulent flows according to numerical simulations^24^ but no experiments exist so far. Rotation effects have also been studied in turbulent Rayleigh Bènard convection and a number of effects have been observed^25^ such as the enhancement of heat transport and its eventual decrease for high rotation rates^26^ as well as the existence of transitions between different turbulent states^27^. A novel aspect of the experiments described here is that the cell used has the advantage of allowing the full hemisphere to be subjected to a global rotation as shown in Fig. 1^28^. Its quasi 2D nature provides possible parallels with large scale atmospheric and geophysical flows and allows simple measurements of the temperature field, a difficult task in 3D cells.

Our diagnostics of the effects of rotation on the turbulent thermal convection is based on the so called temperature structure functions (temperature differences between points separated by a distance *r*, raised to a power *n* and averaged over space and time) extracted from temperature fields on the surface of the bubble. In the absence of rotation, these structure functions follow a scaling law versus *r*, expected and found in earlier experiments^16^ namely Bolgiano-Obukhov scaling, with exponents of *n*/5 for the variation of the n^*th*^ order moments of temperature differences across a scale *r*. For low rotation rates, this scaling barely changes. However, and above a certain threshold in rotation rate, the scaling exponents change and show an *n*/2 behavior. While no predictions exist for such a change, other experiments and phenomenological models have uncovered a similar transition for the velocity structure functions in rotating 3D hydrodynamic turbulence. The transition we find here, although bears the same features, is for temperature fluctuations in turbulent thermal convection so our results are new. We are not aware of other experiments where the effects of rotation on a scalar field are measured, but numerical simulations suggest a different scaling behavior so our results are at odds with expectations in 3D. The role of rotation is thus different in our geometry. Our own numerical simulations carried out for comparable conditions as the experiments suggest a similar result as our experiments namely a transition from n/5 to n/2 as found experimentally. Further increase in rotation rate shows another transition to exponents which increase with the rotation rate suggesting that for high rotations a new regime sets in. That rotation changes scaling exponents of turbulent fluctuations, be they in the hydrodynamic case or in the thermal convection case as shown here, has important consequences on the understanding of the energy content of such fluctuations across the scales, a question which is important in understanding energy transfers in a variety of situations^29^.

## Results

The convection cell, described previously in^28^ and in^16^, is shown in Fig. 1. Briefly, it consists of an outer hollow brass ring, which is heated using water from a thermostat, and a circular inner disk with a groove on its outer periphery on which the bubble is blown. The inner disk is heated by the proximity of the outer ring. The groove on the upper side of the inner disk contains the soap solution (0.5 to 2% liquid detergent in water) which is heated to the desired temperature. The central part of the inner disk is made of a thick black Teflon disk to minimize heating of the air inside the bubble. Half a soap bubble, of 11 cm in diameter, is blown using the soap solution in the groove. The temperature difference between the equator and the pole of the bubble was measured using thermocouples in air. This difference noted ΔT can be varied up to 43 °C. This inner disk is connected to a continuous motor via a shaft. The motor is capable of rotating at different and stable rotation frequencies *f* going from less than 0.1 Hz up to 3 Hz. The limited frequency range is not due to the motor but to the fact that above 3 Hz, the liquid in the groove is ejected through centrifugal forces. Direct measurements of the mean rotation velocity at different latitudes (see Supp. Mat.) shows that the rotation of the bubble introduces a first layer near the equator and extending up to about 10° above the equator where the local rotation rate decreases from its imposed value at the equator. At latitudes above roughly 15°, the local rotation rate remains constant and near 1/3 of the imposed rotation rate. Our measurements are carried out in the region above the first layer and for which the rotation rate is independent of latitude. In this region, the effects of the surrounding air on the dynamics of the flow on the surface of the bubble are estimated to be small (see Supp. Mat.). The temperature field measurements used a calibrated 14 bit infrared camera (640 × 512 pixels) working in the spectral range 2.5 *μm* to 5 *μm* with a sensitivity of 20 *mK* and an adjustable exposure time between 0.5 and 1 ms. The camera can also be fitted with different lenses to vary the spatial resolutions as well as different band pass filters allowing to work in conditions where variations of the emissivity of the soap film constituting the bubble due to variations in thickness are minimized (see Supp. Mat.). Images of the same region (typically of 1.5 × 1.5 cm) (several hundred images at rates between 10 to 100 frames/second) were recorded and used to calculate temperature differences across different scales *r*. Averaging over the area of interest and over hundreds of images allowed us to improve the statistics (between 1 and 5 million points were used) and calculate the moments of these differences. The temperature field was recorded for periods of up to 30 s which is greater than the temperature correlation time (of order 0.1 s) and a few times the smallest rotation times considered. The error in *r*, introduced by the curved geometry of the bubble, is less than a few percent over a 1 cm region.

Figure 1 shows images of the bubble. One can easily identify thermal plumes rising from the bottom of the cell for the low temperature gradients. The thermal convection becomes more intense as the temperature gradient increases and well defined thermal plumes are difficult to discern. The effect of rotation can be seen in Fig. 1 where the thermal plumes are clearly tilted: The higher the rotation rate, the greater the tilt.

From such spatial images, and besides the qualitative aspects just described, we extract the temperature difference *δT*(*r*_*x*_) = *T*(*x* + *r*_*x*_) − *T*(*x*) and *δT*(*r*_*y*_) = *T*(*y* + *r*_*y*_) − *T*(*y*) and calculate the n^*th*^ order moments as ${S}_{n}^{T}({r}_{x})=\langle {|\delta T({r}_{x})|}^{n}\rangle$ and ${S}_{n}^{T}({r}_{y})=\langle {|\delta T({r}_{y})|}^{n}\rangle$ in small areas lying midway between the pole and the equator. Here *x* and *y* refer to the horizontal and vertical coordinates and the brackets refer to an average over space and time. For simplicity, we will focus on the second moment n = 2 for the rest of the discussion. Structure functions are important quantities in the study of turbulence and different scaling relations have been proposed for their variation versus the scale *r*. In 3D turbulent flows, where Kolmogorov scaling for the velocity fluctuations is believed to prevail, Obukhov and Corrsin^5,6^ generalized the scaling arguments of Kolmogorov to a scalar field like the temperature and used both the energy dissipation rate *∈* and the scalar dissipation rate *∈*_*T*_ to predict that the n^*th*^ order structure functions should scale as ${\in }_{T}^{n\mathrm{/2}}{\in }^{-n\mathrm{/6}}{r}^{n\mathrm{/3}}$. Similar scaling arguments can be used, as suggested by Bolgiano and Obukhov^9–11^ for stably stratified turbulence, to the case of Rayleigh Bénard convection for which the fluid thermal expansion rate *β*, the gravity constant *g*, and the dissipation rate *∈*_*T*_ fix the scaling relation of the n^*th*^ order structure function of the temperature as ${\in }_{T}^{2n\mathrm{/5}}{(\beta g)}^{-n\mathrm{/5}}$ *r*^*n*/5^ ^9^. The n^*th*^ order moments are thus expected to vary as a power law of the separation distance *r* with an exponent ${\zeta }_{n}^{T}$ of *n*/5 in the Bolgiano-Obukhov regime. To compare the experimental conditions here to their classical counterparts, we estimate the Rayleigh number (*Ra* = *β*Δ*TgR*^3^/*νκ* where *ν* and *κ* are the kinematic viscosity and the thermal diffusivity of water) to be between 7 10^7^ and 3 10^8^ while the Reynolds number (*Re* = *V*_*mean*_*R*/*ν* where *V*_*mean*_ is the characteristic horizontal velocity) is estimated to be about 3000^16^. In previous experiments using the soap bubble, and for Δ*T* > 35 °*C*, the exponent ${\zeta }_{n}^{T}$ turned out to be close to *n*/5^16^ strongly suggesting the presence of a Bolgiano-Obukhov scaling. Here we examine how this scaling behavior changes with the global rotation of the bubble.

To gauge the effects of rotation, a non dimensional number, the so called Rossby number *Ro* is introduced: *Ro* compares the advection to rotation time scales and is generally written as *Ro* = *V*/Ω*R* where *V* is a characteristic velocity, *R* a characteristic length (the radius of the bubble in our case) and Ω = 2*πf* is the angular rotation rate. For *Ro* values greater than 1, rotation plays a minor role but when *Ro* reaches values near 1 and smaller, rotation effects on the properties of the flow are expected to become important. This number can be recast, for thermal convection driven flows as $Ro=\frac{1}{2{\rm{\Omega }}}\sqrt{\frac{\beta {\rm{\Delta }}Tg}{R}}$^26,30^. In the experiments described here *Ro* is varied down to values near 0.1.

**Figure 2:**
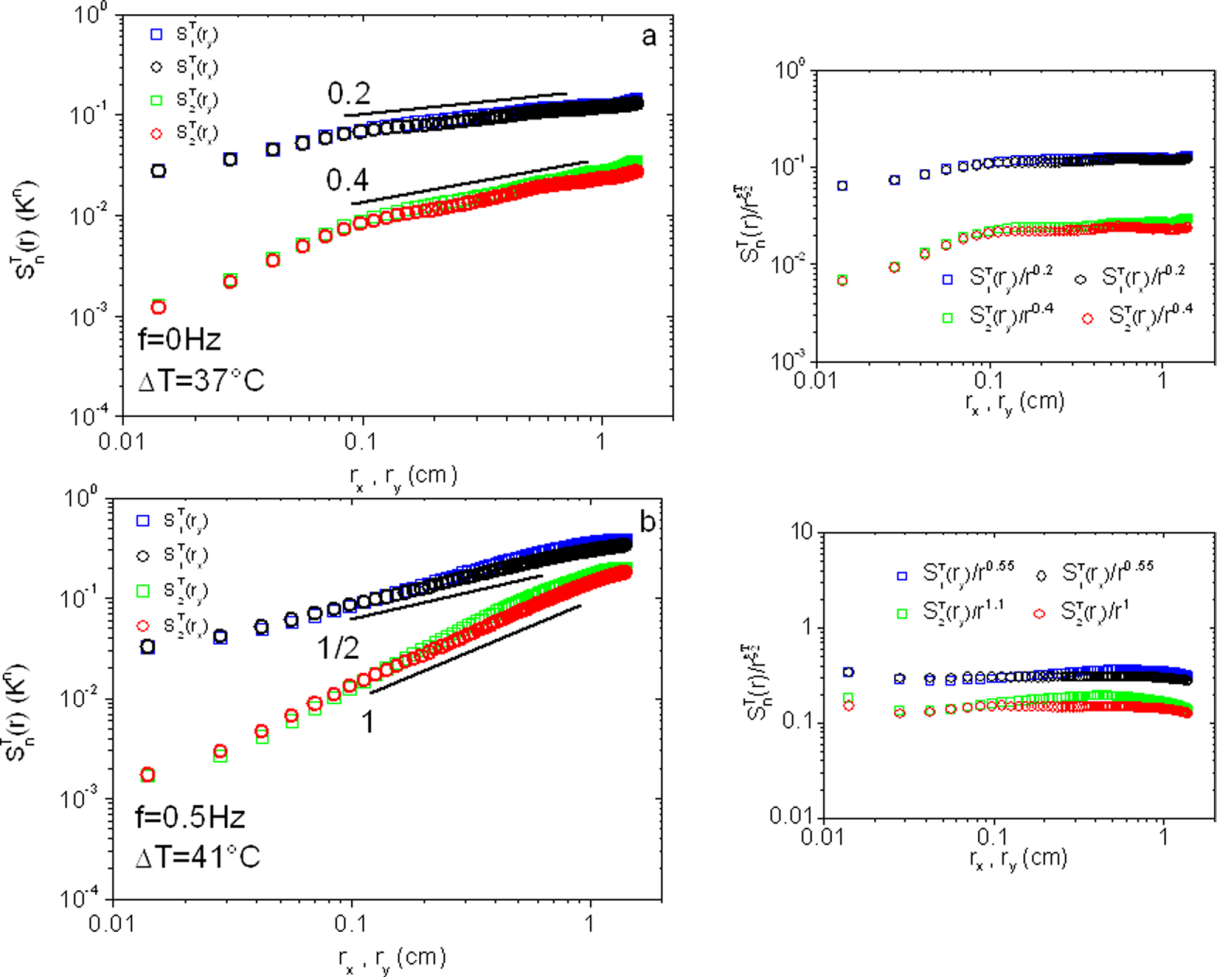
Temperature structure functions for a) Δ*T* = 37.5 °C at 0 Hz and b) Δ*T* = 41 °C at 0.5 Hz. Insets: Compensated moments with the exponents used given in the legends.

Rotation effects are studied for temperature gradients >35° where the scaling of the temperature structure functions follows the Bolgiano-Obukhov regime. The structure functions of order 1 and 2 are displayed in Fig. 2a,b. For no rotation or for low Ω, Fig. 2a, the temperature structure functions are roughly isotropic as their values for the two orthogonal spatial increments *r*_*x*_ and *r*_*y*_ are similar. These functions display power law scaling for spatial scales between roughly 1 mm and 10 mm. The scaling exponents are in agreement with predictions of Bolgiano and Obukhov as reported before^16^.

The results for the structure functions in the presence of rotation are shown in Fig. 2b. These functions again show isotropy and power law scaling versus *r*. The exponents turn out to be different from the above results. The scaling is observed in the range 1 to 10 mm as shown in Fig. 2b which displays the compensated moments in the graph to the right. Instead of the n/5 (i.e. 0.2 and 0.4 for the moments of order 1 and 2 respectively) observed without rotation, an n/2 (i.e. 0.5 and 1 for moments of order 1 and 2) variation seems to prevail. Note here that Ω/2*π* needs to be large enough (>0.1 Hz) for the transition from n/5 to n/2 behavior to occur. This corresponds to an *Ro* of roughly 1. Further increase in the rotation frequency, and therefore a decrease in the value of *Ro*, did not change the exponents. However, and for higher rotation frequencies such that *Ro* decreases below 0.25, a new increase in the value of ${\zeta }_{2}^{T}$ sets in. This increase seems continuous with ${\zeta }_{2}^{T}$ reaching values near 2 as we will see below.

**Figure 3:**
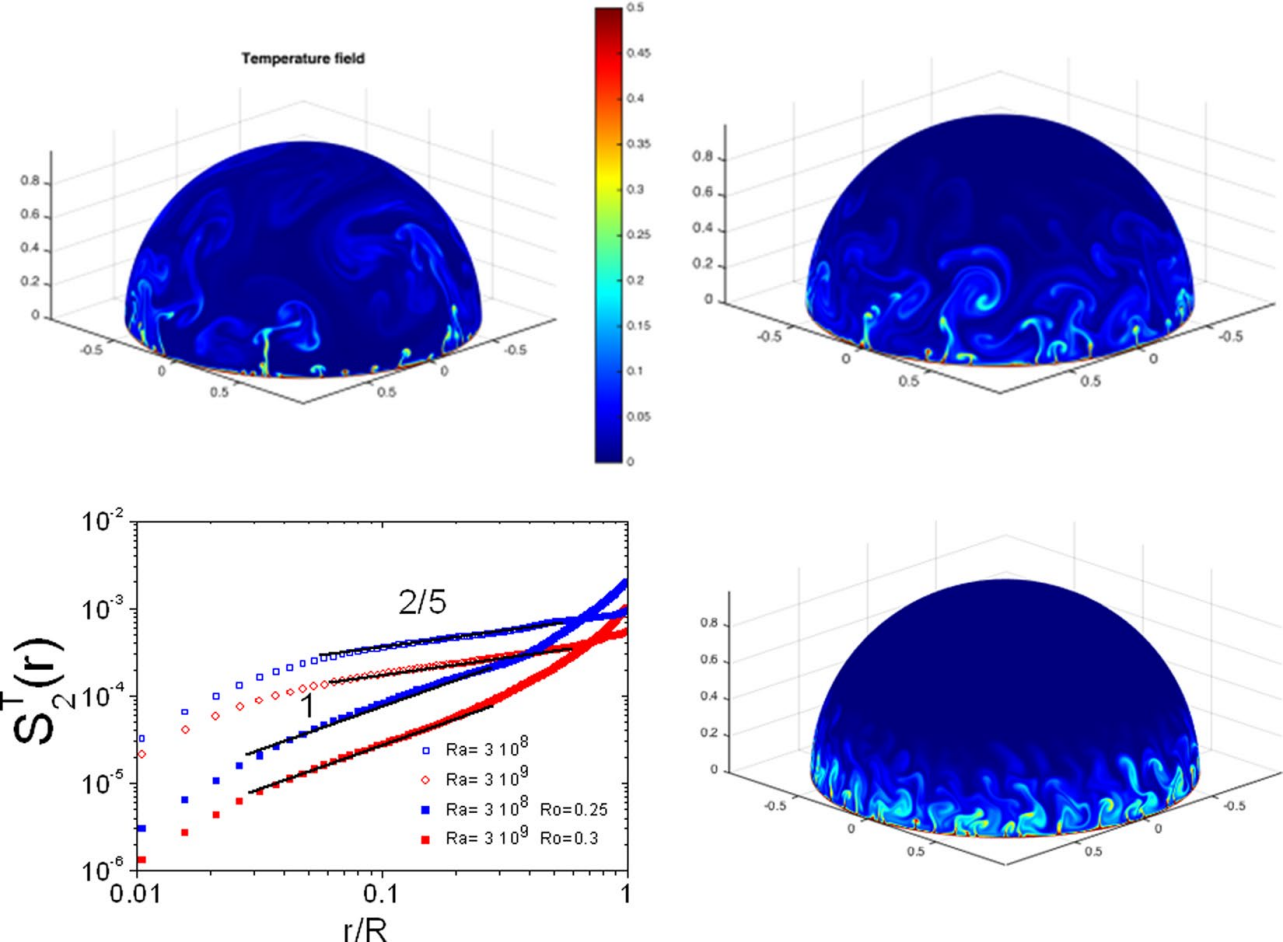
Images of the temperature field and plots of the second order structure functions with and without rotation from DNS. The horizontal scale has been normalized by the bubble radius R. Note that the scaling range between 0.04R and 0.3R corresponds roughly to the scaling range observed in experiments which extends between 0.02R and 0.2R (R is 5.5 cm in experiments). The black lines indicate the scalings observed in experiments namely 2/5 for the Bolgiano scaling and 1 under rotation. The photos correspond to (from upper left to bottom right): Ra = 3 10^8^ for no rotation, Ro = 0.25 and Ro = 0.05 respectively.

The experimental results presented here are confronted to the results of direct numerical simulations (DNS) of thermal convection in a hemispherical geometry as explained in^28,31,32^. Briefly, a hemisphere of radius 1, subjected to thermal convection is described by the coupled Navier -Stokes and the heat equation in the Boussinesq approximation which are simulated using a stereographic projection. The results of these simulations are shown in Fig. 3 where images of the temperature field on the surface of the bubble are shown along with the second order moment of temperature differences. The main message of this figure is that the Bolgiano scaling observed experimentally is obtained for no rotation confirming the robustness of our experimental results. When rotation is introduced, a steepening of the second order structure function, at scales comparable to the range where experiments show a higher exponent, is then observed. This steepening is consistent with the increase of the exponent observed experimentally as an exponent near 1 is observed for two different *Ra* numbers and for *Ro* values smaller than 1 and comparable to those of the experiments. As *Ro* decreases below 0.25, the exponent ${\zeta }_{2}^{T}$ starts to increase again and adopts values higher than 1 as will be discussed below.

**Figure 4:**
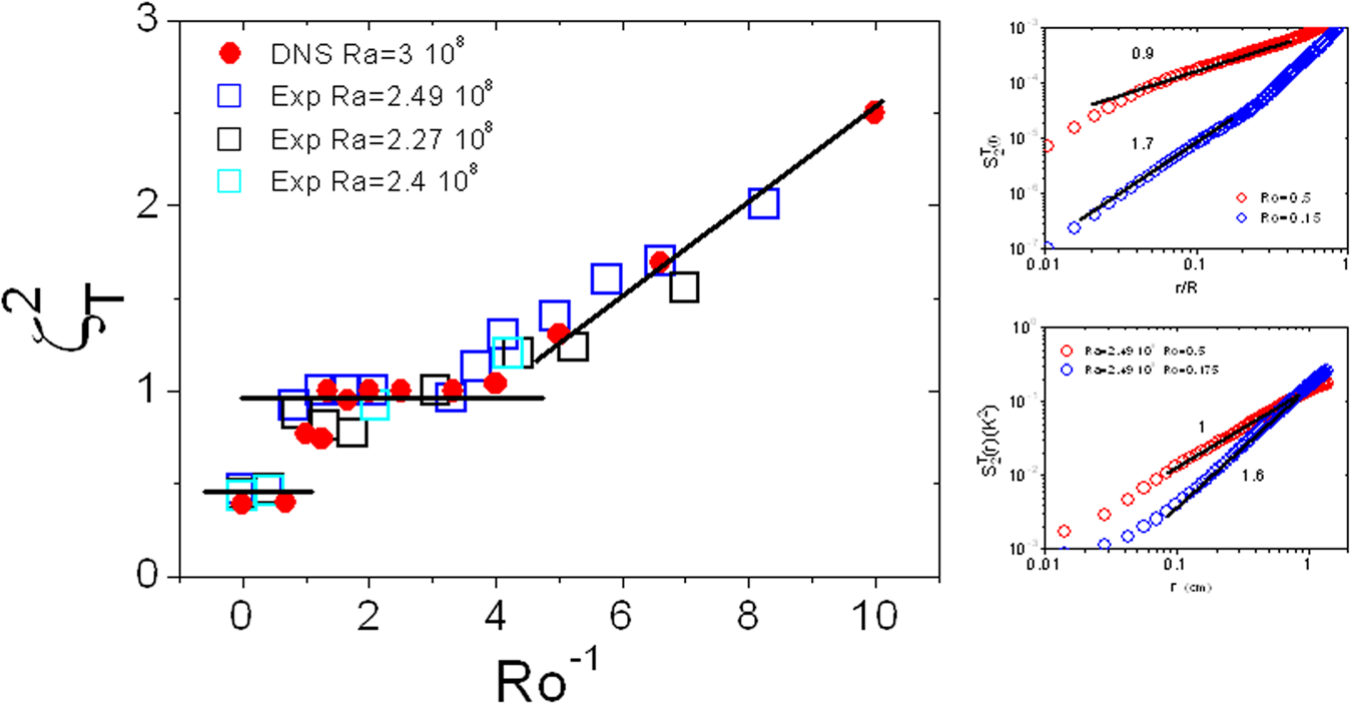
${\zeta }_{2}^{T}$ versus *Ro*^−1^ for the numerical and experimental data. The insets compare structure functions at different Ro for numerical simulations at Ra = 3 10^8^ and for experiments. Note that the smaller Ro data have exponents greater than 1.

This behavior and changes in the scaling of the temperature structure functions can be appreciated in Fig. 4 where ${\zeta }_{2}^{T}$ is displayed versus *Ro*^−1^. Note that for *Ro*^−1^ < 1, the exponent is roughly the same as for no rotation. For *Ro*^−1^ > 1 and higher, ${\zeta }_{2}^{T}$ remains close to 1 up to $R{o}^{-1}\simeq 4$. Above this value, the exponent starts to show an increase as *Ro*^−1^ increases: ${\zeta }_{2}^{T}$ increases to values higher than 1 and reaches values greater than 2 for *Ro*^−1^ near 10. The plots on the right in Fig. 4 show examples of the structure functions from numerics and experiments for *Ro*^−1^ > 4 and for which ${\zeta }_{2}^{T}\mathrm{ > 1.}$ Our experimental and numerical results show a transition from Bolgiano Obukhov scaling to a new scaling exponent as the Rossby number reaches a value near 1 where rotation effects become comparable to advection effects. Further and as *Ro* decreases to small values of order 0.1 where rotation effects are supposed to dominate the flow, the scaling exponent becomes even steeper.

## Discussion and Conclusions

So far, only results for the role of rotation on the velocity structure functions in 3D hydrodynamic turbulence have been obtained. Zhou^33^ and Canuto and Dubovikov^34,35^ argued that if Ω^−1^ is used as the characteristic time scale for local energy transfers in rotating hydrodynamic turbulence as suggested in^36^, a scaling in n/2 can be obtained instead of n/3 for the velocity structure functions. Other arguments have been put forth in^37^ who introduces a new length scale determined by the rotation rate and the dissipation rate of the turbulent flow. These arguments mostly rely on the introduction of a new time scale, or a new length scale, both determined by the rotation rate, which change the scaling of the energy density spectra. Experimental evidence of the n/2 scaling has been obtained in^21^ while in^22,23^ a continuous increase of the exponent from n/3 at low rotations rates to steeper and steeper exponents has been observed. For a scalar field such as the temperature studied here, on the other hand, numerical simulations find an equivalent scaling in n/4^24^ when the velocity structure functions scale as n/2. This scaling is consistent with the velocity scaling since both are related by ${\zeta }_{1}^{V}$ + ${\zeta }_{2}^{T}$ = 1 for fully developed turbulence, where ${\zeta }_{1}^{V}$ is the scaling exponent of the first order absolute velocity structure function^24^. Our scaling exponents for n = 2 are clearly higher than the expected exponent both in experiments and in numerics making our quasi two dimensional system with a spherical geometry quite different from its three dimensional counterparts. The above relation between scaling exponents for the velocity and the scalar field suggests that the scaling exponent of the velocity structure function of order 2, in our case for moderate values of *Ro*, should be much smaller than 1 and rather close to zero. This is at odds with the three dimensional expectations and suggests that our geometry and the dimensionality of the experiments (quasi 2D instead of 3D) play an important role in how rotation modifies the statistical properties of turbulent flows. To further check these results we have carried out additional experiments to measure the velocity structure functions. These results show that the velocity structure functions display Bolgiano Obukhov scaling consistent with an exponent of 3n/5 as expected and observed experimentally before^12,13,16^. Under rotation, the scaling range diminishes and the structure function exponents become smaller, contrary to 3D experiments where the exponent increases. This is compatible with the expected exponent from the scalar measurements. In fact, the velocity structure functions are compatible with a logarithmic variation which again comforts the fact that we expect a small scaling exponent if any for the velocity structure functions. These results are shown in Supp. Mat. This result is only valid for ${\zeta }_{2}^{T}\simeq 1$ and therefore for moderate values of *Ro*. In fact, a value of ${\zeta }_{2}^{T}\simeq 1$ is special in that it is reminiscent of a classical random process, namely Brownian diffusion^38^. In this particular case, the statistical properties of the temperature become of a diffusive character while the underlying velocity field loses spatial correlations. In any case, rotation makes for a large decrease of the velocity fluctuations as shown in Supp. Mat.

Further, for *Ro* values smaller than 0.2 the exponent increases to values higher than 1, the relation between ${\zeta }_{1}^{V}$, ${\zeta }_{2}^{T}$ must not be valid any more suggesting that the flow has changed its turbulent character. We have noted in experiments that for high rotation rates, the plumes tend to tilt and have difficulty reaching the pole. In numerics, the smaller the value of *Ro*, the less plumes there are at the pole as shown in Fig. 3. The pole area seems to be devoid of any fluctuations. Rotation inhibits fluctuations at the pole rendering this area ‘laminar’. This effect is related to the Coriolis force since a velocity fluctuation towards the pole will necessarily be deflected confining velocity fluctuations to regions closer and closer to the equator as the rotation rate increases.

Our novel quasi 2D rotating convection cell allows for a detailed study of rotation effects on the statistical properties of temperature fluctuations in turbulent thermal convection. These properties show a transition from Bolgiano-Obukhov scaling to a new scaling regime with nearly n/2 scaling for moderate Rossby numbers. This transition is also obtained in direct numerical simulations. This new scaling is at odds with expected results from theoretical expectations and three dimensional numerical simulations and suggests that the dimensionality of the system used as well as its geometry play a role in setting how rotation affects the statistical properties of turbulent flows. Further decrease in Ro leads to even higher exponents suggesting a re-laminarization of the flow for high enough rotation rates. Considering that our system, which has a natural curvature with quasi two dimensional flow properties, bears similarities with geophysical systems for which rotation governs a number of properties, the results found here call for new thinking about the role of rotation in the modification of turbulence properties.

## Electronic supplementary material

Supplementary material
