# Supplementary material for: Effects of rotation on temperature fluctuations in turbulent thermal convection on a hemisphere
Source: Sci Rep. 2018 Nov 8;8:16513. doi: 10.1038/s41598-018-34782-0 (PMC6224521; doi:10.1038/s41598-018-34782-0)
Supplement: Supplementary file 1 — Supplementary material [file 41598_2018_34782_MOESM1_ESM.pdf]

Effects of rotation on temperature fluctuations in turbulent thermal convection on a hemisphere

T. Meuel, M. Coudert, P. Fischer, C. H. Bruneau, and H. Kellay

This file presents supplementary figures and information for the main paper.

I. **Measurements of the local rotation rate:**

One of the important features of our system is its ability to rotate the turbulent system globally. This rotation is imposed at the equator of the half bubble where it is in contact with the hot water reservoir. In order to determine the local rotation rate on the surface of the bubble, we have measured the rotation velocity at different latitudes. The figure Supp. Fig. 1, shows the local rotation rate, normalized by the imposed rotation rate versus latitude. Different imposed rates are used. This figure shows that the rotation rate is not constant over the whole surface of the bubble. In the layer near the equator, up to roughly 10°, the local rotation decreases from its imposed value. Above roughly 15° in latitude, the local rotation rate seems to stabilize on a plateau at roughly ¼ of the imposed rotation rate. This plateau persists up to latitudes beyond 60°. Our measurements of the temperature field were carried out in this region (near 45°) where the rotation rate is roughly constant and independent of latitude. It is this measured rate which is used to calculate the Rossby number.

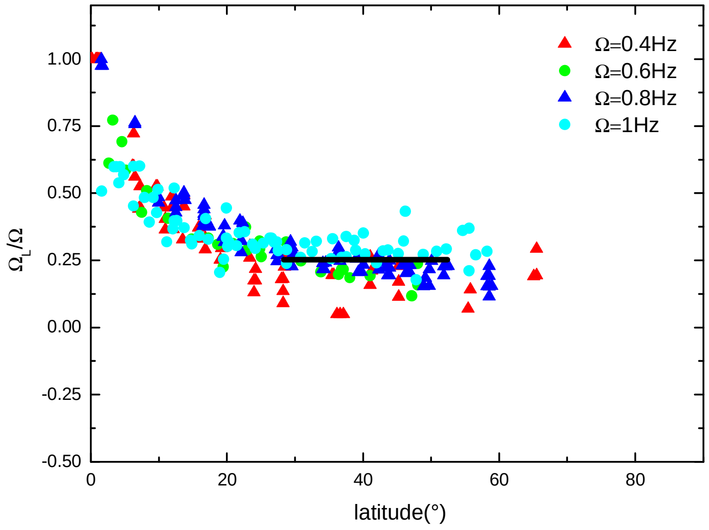

Supp. Fig 1 : Measured rotation rate versus latitude for different imposed rotation rates Ω

II. **Air effects:**

The bubble rotates in air. The entrainment of the surrounding air by the bubble may affect the influence of rotation on the statistics of the turbulent flow through the shearing of the air layer. Two estimates can be made to gauge this effect. Let us first estimate the so called Ekman number which compares the viscous effects in air to the rotation effects. This number

is written as $Ek=\nu/\Omega L^2$. Where ν is the kinematic viscosity of air, Ω is the angular rotation, and L a characteristic length scale. An estimate of this number using an imposed rotation frequency of 1Hz and a characteristic length of 1cm gives 10^{-3}. Clearly rotation effects are important with respect to viscous effects. A second estimate to gauge this effect is to compare the shear stress in the surrounding air to the turbulent stresses in the film. Let us use a conservative estimate of the velocity fluctuations of 1cm/s and by setting the turbulent stress estimate as $\rho\, v_{rms}^2$ we find 10^{-1} Pa. if we consider the velocities at the surface of the bubble and a shear layer thickness in the air of about 1mm, the shear stress in air is 10^{-3} Pa. Both estimates indicate that the air is most probably playing a minor role in setting the influence of rotating the cell on the change in exponents we observe.

Further, this change seems to occur abruptly, near a Rossby number of 1 where rotation effects and advection effects become comparable which is difficult to reconcile with the progressive effect of air as the rotation increases.

III. Velocity structure functions.

As mentioned in the main text, our findings for the temperature structure functions has repercussions on the scaling of the velocity structure functions through the relation $\zeta_1^V+\zeta_2^T=1$ where ζ_1^V is the scaling exponent of the first order structure function of the velocity and ζ_1^T is the scaling exponent of the first order structure function of the temperature. This relation is valid only for the case where the temperature injection or dissipation rate is constant. From the scaling found in the main text, and if we assume that this injection rate is constant, we expect ζ_1^V to be near zero indicating a possible logarithmic variation of the velocity structure function.

We have therefore carried out Particle imaging velocimetry in similar regions as for the temperature measurements of the velocity field on the bubble surface. The structure functions obtained are shown in Supp. Fig 2. Note that for no rotation, Supp. Fig. 2a, the scaling of the 1st and 2^{d} order structure functions is consistent with the expected Bolgiano Obukhov scaling in 3n/5. Rotation changes this scaling exponent and makes for structure functions which vary much more slowly. The quality of a power law scaling, if any, turns out to be modest, less than a decade, as seen in Supp. Fig 2b for a rotation frequency of 0.4Hz. The bottom figure Supp Fig 2c shows that the structure function of order 1 (of Supp. Fig. 2b) is rather compatible with a logarithmic variation comforting our temperature results which suggest that the velocity structure functions should have a scaling exponent near zero for this range of rotation rates. A final remark concerns the amplitude of fluctuations of the velocity. It should be noted that this amplitude decreases markedly as the rotation is introduced. For the case of Fig. 2, the change in fluctuation amplitude is nearly tenfold. The velocity fluctuations are clearly inhibited by rotation. In Fig. 2 d, the standard deviation of the fluctuations of the velocity from numerical simulations conmforts these observations as the fluctuations drop by two orders of magnitude for Ro values smaller than 1.

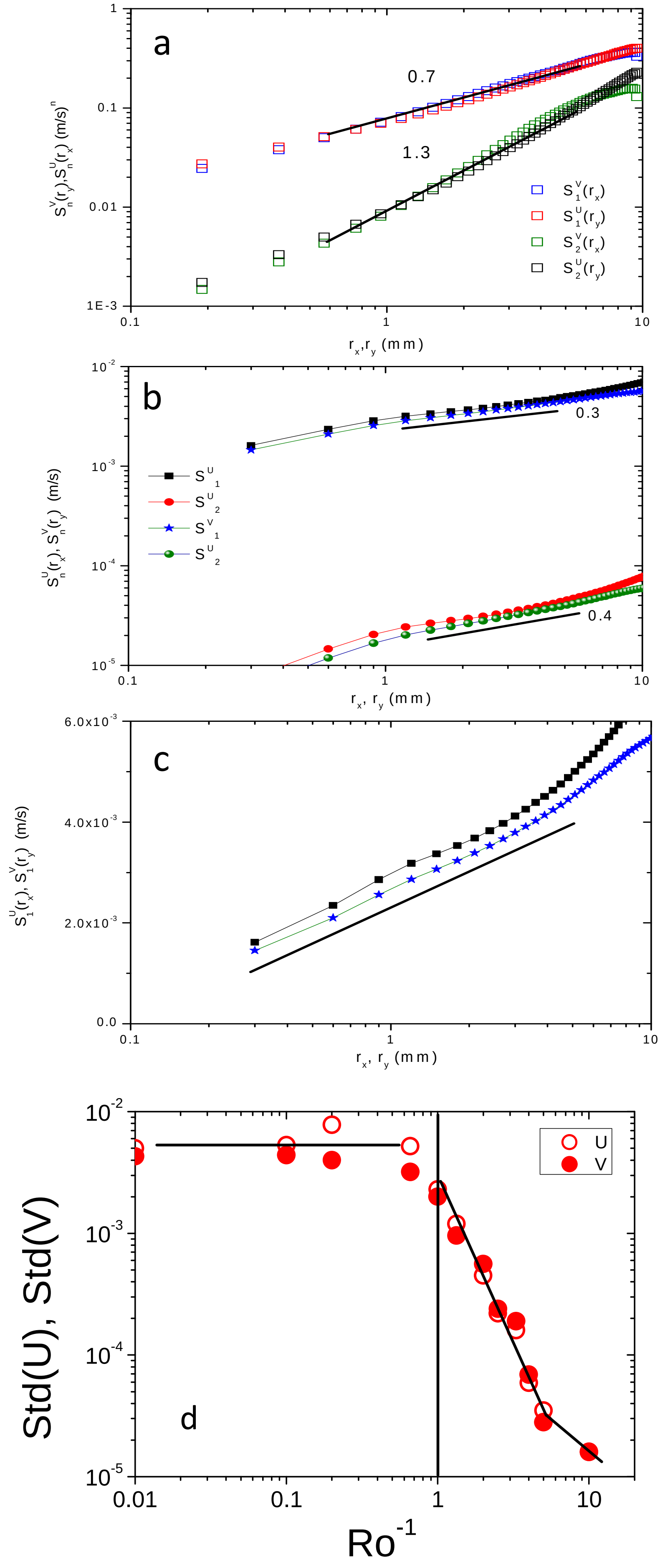
a
0.7
1.3
$S^V_1(r_x)$
$S^U_1(r_y)$
$S^V_2(r_x)$
$S^U_2(r_y)$
$S^V_n(r_y), S^U_n(r_x)$ (m/s)^n
r_x, r_y (mm)
b
0.3
0.4
S^U_1
S^U_2
S^V_1
S^U_2
$S^U_n(r_x), S^V_n(r_y)$ (m/s)
r_x, r_y (mm)
c
$S^U_1(r_x), S^V_1(r_y)$ (m/s)
r_x, r_y (mm)
d
U
V
Std(U), Std(V)
Ro^{-1}

Supp. Fig. 2: For no rotation a) the scaling of the 1st and 2d order structure functions is consistent with the expected Bolgiano Obukhov scaling in 3n/5. b) Rotation changes this scaling exponent which becomes smaller, the rotation frequency is 0.4Hz. c) shows that the structure function of order 1 (of Supp. Fig. 2b) is rather compatible with a logarithmic variation. d) Standard deviation of velocity fluctuations in the latitude and longitude directions versus Ro^{-1}

IV. Infrared images of soap films and temperature measurement:

Infrared images of a soap film as for our bubble pose the problem of what the emissivity of the films is. The intensity I of the signal received by the camera can be written as $I=\varepsilon T^4$.

Here ε is the emissivity of the body and T its temperature. The soap film being constituted of 99% water, it can be considered as a slab of water with a certain thickness h. In our case this thickness is of a few microns. In this case and for a thickness h of the film, the emissivity can be written as $\varepsilon = 1-\exp(-h/Z_0(\lambda))$ (see J. Zhang, X. L. Wu, and N. Rashidnia, PoF 18, 085110 (2006) in the main text)

Z_0 is the penetration length of the infrared radiation of wavelength λ. Ideally, one wants an emissivity which is 1 or close to 1 and insensitive to thickness variations to measure the temperature on the surface of the soap bubble.

We have tested that the thickness does not influence the results by using different band pass filters on the camera. For each filter the value of Z_0 is different giving different weights to the exponential factor above. The results are shown in Supp. Fig 3.

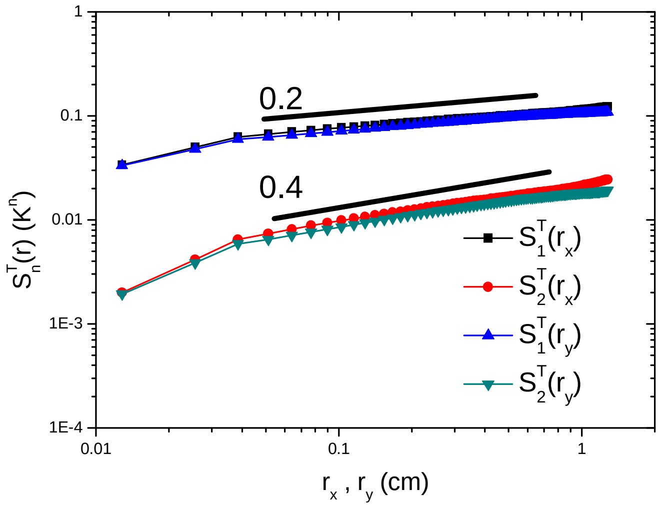

Supp. Fig 3a: Temperature structure functions using a filter working in the wavelength range 2.9 to 3.5 microns

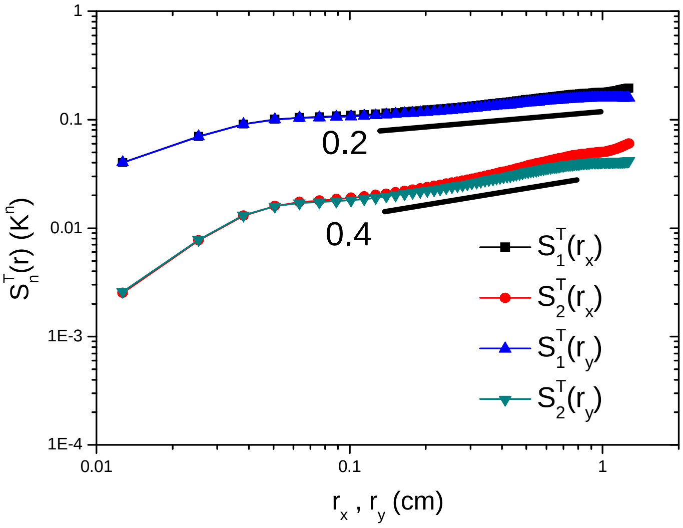

Supp. Fig 3b: Temperature structure functions using a filter working in the wavelength range 2.95 to 3.26 microns

Note that for each width of the filter, the penetration length Z_0 is different. It is of 1.1 microns for the filter in the range 2.95 to 3.26 microns and it is 1.6 microns for the filter in the range 2.9 to 3.5 microns. These lengths were measured using transmission measurements on vertical soap films. Both the measured lengths are smaller than the mean thickness of the films which is roughly 3 microns making the exponential term in the emissivity small with respect to 1. The important observation though is that the scaling exponent is independent of the working wavelength range and is similar to the one measured without using a filter. The data shown in the main text do not use a filter.

These observations, along with the fact that the measured exponent for second order temperature structure functions is also obtained in numerical simulations and is very different from the observed scaling of the thickness field (see references Seychelles et al, PRL 2008, and Zhang and Wu PRL 2005 in the main text) comfort us in our use of infrared thermography to measure temperature fluctuations in soap films.
